# *Bacillus amyloliquefaciens* B10 Alleviates the Immunosuppressive Effects of Deoxynivalenol and Porcine Circovirus Type 2 Infection

**DOI:** 10.3390/toxins16010014

**Published:** 2023-12-27

**Authors:** Huiping Xiao, Zihui Qin, Baocai Xu, Miao Long, Qinghua Wu, Xinyi Guo, Huayue Zhang, Zelin Li, Wenda Wu

**Affiliations:** 1 Joint Research Center for Foodborne Functional Factors and Green Preparation, School of Food and Biological Engineering, Engineering Research Center of Bio-Process, Ministry of Education, Hefei University of Technology, Hefei 230009, China; huiping0729@163.com (H.X.); 2019107104@njau.edu.cn (Z.Q.); baocaixu@163.com (B.X.); 2021107103@stu.njau.edu.cn (X.G.); huayuez163@163.com (H.Z.); zelinli0131@163.com (Z.L.); 2 MOE Joint International Research Laboratory of Animal Health and Food Safety, College of Veterinary Medicine, Nanjing Agricultural University, Nanjing 210095, China; 3 Department of Chemistry, Faculty of Science, University of Hradec Kralove, 50003 Hradec Kralove, Czech Republic; wqh212@hotmail.com; 4 Key Laboratory of Zoonosis of Liaoning Province, College of Animal Science & Veterinary Medicine, Shenyang Agricultural University, Shenyang 110866, China; longmiao@syau.edu.cn; 5 College of Life Science, Yangtze University, Jingzhou 434025, China

**Keywords:** *Bacillus amyloliquefaciens* B10, deoxynivalenol, porcine circovirus type 2, autophagy, cGAS–STING

## Abstract

As one of the most common mycotoxins, deoxynivalenol (DON) can contaminate a wide range of crops and foods. Porcine circovirus 2 (PCV2) is a kind of immunosuppressive virus, which can cause porcine circovirus associated disease (PCVD) in pig farms infected with PCV2. Pigs are extremely sensitive to DON, and PCV2-infected pig farms are often contaminated with DON. Our previous studies indicated that *Bacillus amyloliquefaciens* B10 (B10) has the potential to alleviate the toxicity of mycotoxins. The research was aimed at investigating the effects of *Bacillus amyloliquefaciens* B10 on the immunosuppressive effects caused by both DON and PCV2 infection. The results indicated that the expression of the PCV2 capsid protein CAP was significantly decreased after pretreatment with *Bacillus amyloliquefaciens* B10. Then, the effects of the *Bacillus amyloliquefaciens* B10 pretreatment on the type I interferon, antiviral protein and the antiviral signal pathway cGAS–STING was further investigated. The findings displayed that the expression of the type I interferon and antiviral protein were increased, while the IL-10 were decreased after pretreatment with *Bacillus amyloliquefaciens* B10. The inhibition of DON on the cGAS–STING signal pathway was relieved. Furthermore, it was found that this intervention effect was produced by inhibiting autophagy. In summary, *Bacillus amyloliquefaciens* B10 can mitigate the immunosuppressive effects of PCV2 and DON by inhibiting the production of autophagy.

## 1. Introduction

As one of the most common mycotoxins found in feed, DON belongs to a type B trichothecene mycotoxin and can contaminate almost all agricultural and sideline products, mainly grain-based food crops such as wheat, corn, and barley [1,2]. DON is not only widely found in grains and feeds, but also widely exists in animal organisms after these grain feeds are eaten by animals and absorbed by the body. It can cause a succession of adverse reactions, including vomiting, diarrhea, anorexia, growth retardation, and immunosuppression. Specifically, DON-induced immunosuppression is of noteworthy concentrate on animal health [3].

Porcine circovirus (PCV) is a single-stranded, circular and unenveloped DNA virus, which is the smallest known animal virus [4]. PCV2 is widely prevalent worldwide and can cause a variety of clinical symptoms, such as postweaning multisystemic wasting syndrome, porcine dermatitis and nephropathy syndrome, and pork granulomatous enteritis [5]. Although PCV2 vaccines have been developed, this virus still infects pigs due to its immunosuppressive effects. Pigs are extremely sensitive to DON, and PCV2-infected pig farms are often contaminated with DON. Since DON also has the ability to cause immunosuppression, it is possible that pigs will be affected by the combined effect of PCV2 and DON [6,7].

*Bacillus amyloliquefaciens*, a genus of Bacillus, is gram-positive and short-rod-shaped [8,9]. There are currently 273 species of *Bacillus amyloliquefaciens* identified [9]. *Bacillus amyloliquefaciens* has antiviral, antitumor, anticancer, immunomodulatory, and antioxidant activities [10]. *Bacillus amyloliquefaciens* has the ability to degrade aflatoxin, ochratoxin, and zeara-lenone [11,12,13]. Previous studies have shown that *Bacillus amyloliquefaciens* B10 had the ability to antagonize AFB1-induced oxidative stress and apoptosis, enhance the antioxidant capacity of the host kidney and liver, and enhance the function of both [14,15]. Additionally, *Bacillus amyloliquefaciens* B10 was able to improve the intestinal barrier and regulate the intestinal microbiota in mice, thereby inhibiting aflatoxin B1-induced cecal inflammation in mice [16].

Cyclic guanosine monophosphate-adenylate synthase (cGAS) is a cell receptor that mainly identifies double-stranded DNA and stimulates innate immune responses, including inducing the expression of interferon [17]. It activates the interferon stimulating factor STING. The cGAS–STING pathway has turned into a crucial mechanism for combining DNA-derived substances with powerful innate immune defense processes [18]. In fact, the cGAS–STING signaling pathway is closely related to autophagy, which not only induces autophagy as a cellular autonomic defense mechanism but is also regulated by autophagy components [18]. Autophagy also plays an important role in natural immunity, which has a “double-edged sword” function according to the cycle of pathogen invasion and replication [19]. On the one hand, it plays an antiviral role in viral infection. On the other hand, it can be undesirably activated in the body’s immunosuppression, thereby promoting viral infection. Nassour’s research found that as the virus replicates, cells participate in the STING–autophagy pathway to drive the autophagic cell death program, thereby preventing tumor cell growth [20]. However, autophagy in the pathological state can, in turn, inhibit the antiviral effects of this pathway [21]. LC3B was detected to determine the occurrence of autophagy, thus indicating the changes in this pathway.

In order to study the role of *Bacillus amyloliquefaciens* B10 in detoxification and antiviral activity, a model of porcine kidney cells (PK-15) infected by PCV2 was established. Using this model, we aimed to explore the role of *Bacillus amyloliquefaciens* B10 on the PK-15 cell immune function in combination with PCV2 and DON. We hypothesized that *Bacillus amyloliquefaciens* B10 would interfere with the immune-lowering effects of PCV2 in combination with DON through autophagy. The fundings of this study will provide novel insights into the targeted therapy of PCV2 and DON infection.

## 2. Results

### 2.1. Cell Viability Assay

**Figure 1:**
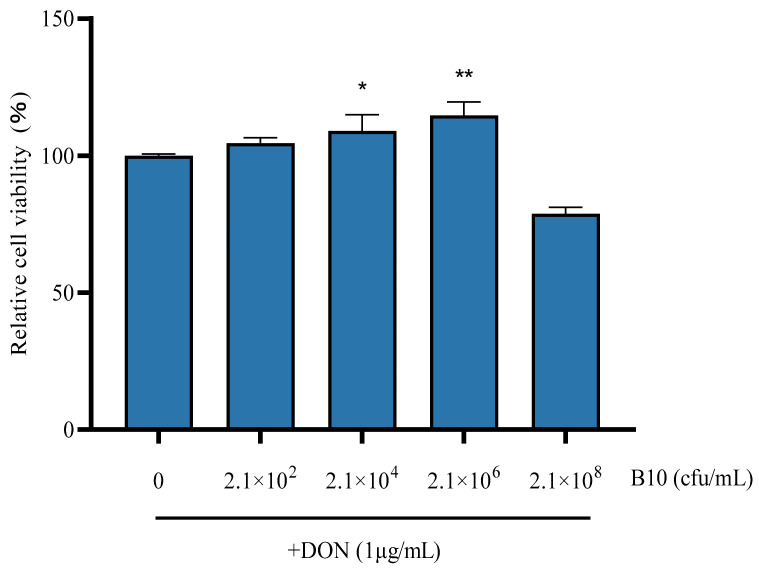
Effect of *Bacillus amyloliquefaciens* B10 pretreatment on cell viability. The data for each assay were analyzed using a one-way analysis of variance (ANOVA). The data were expressed as the mean ± SD (*n* = 6). Compared with the control group, * *p* < 0.05 indicated a significant difference and ** *p* < 0.01 indicated a very significant difference.

Compared with the control group, cell viability was significantly reduced in the *Bacillus amyloliquefaciens* B10 group at 2.1 × 10^8^ cfu/mL. The other groups alleviated the damage to the cell, and the 2.1 × 10^6^ cfu/mL group significantly alleviated the damage of the PK-15 cells caused by DON-upregulated PCV2 infection (Figure 1). Therefore, a concentration of 2.1 × 10^6^ cfu/mL *Bacillus amyloliquefaciens* B10 was used for the follow-up experiments.

### 2.2. Screening of the Pretreatment Time of Bacillus amyloliquefaciens B10

**Figure 2:**
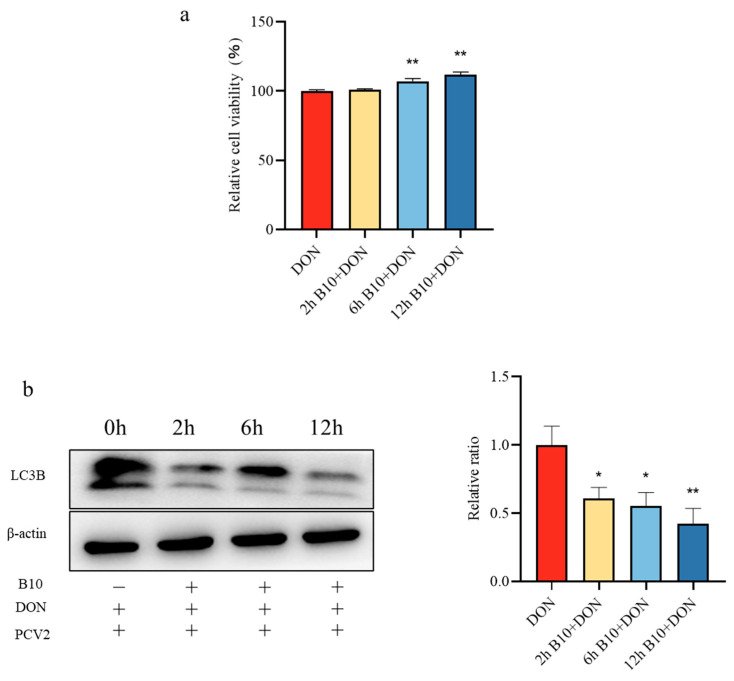
Optimal treatment time for *Bacillus amyloliquefaciens* B10. (**a**) The optimal treatment time of *Bacillus amyloliquefaciens* B10 was determined by the MTT assay; (**b**) the effective treatment time of *Bacillus amyloliquefaciens* B10 was verified by detecting the expression of the LC3B protein. The data for each assay were analyzed using a one-way analysis of variance (ANOVA). The data were expressed as the mean ± SD (*n* = 3). Compared with the control group, * *p* < 0.05 indicated a significant difference and ** *p* < 0.01 indicated a very significant difference.

In order to determine the optimal treatment time, an MTT assay and Western Blot assay were used. Compared with the control group, *Bacillus amyloliquefaciens* B10 pretreatment for 2 h, 6 h, and 12 h significantly alleviated the damage caused by DON to the cells, and the effect of the *Bacillus amyloliquefaciens* B10 pretreatment at 12 h was better (Figure 2a). Meanwhile, the expression level of the LC3B protein was significantly decreased at different pretreatment times (Figure 2b), and the expression of the LC3B protein was reduced more significantly after 12 h of *Bacillus amyloliquefaciens* B10 pretreatment. Therefore, *Bacillus amyloliquefaciens* B10 was pretreated for 12 h for the subsequent experiments, and *Bacillus amyloliquefaciens* B10 exposure induced autophagy.

### 2.3. Effect of Bacillus amyloliquefaciens B10 on DON Promoting PCV2 Infection

**Figure 3:**
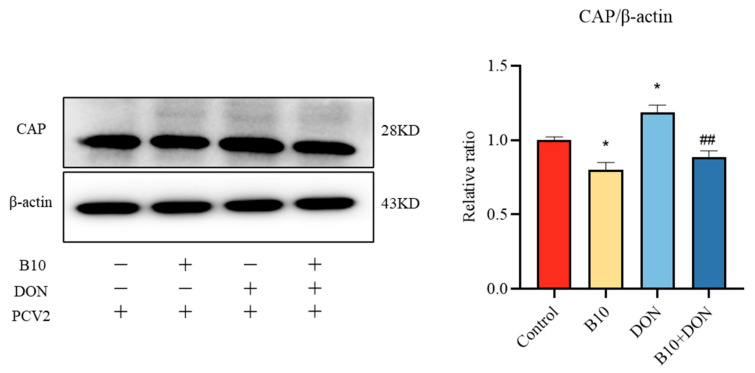
Effect of DON on PCV2 infection in vitro after *Bacillus amyloliquefaciens* B10 pretreatment. The data for each assay were analyzed using a one-way analysis of variance (ANOVA). The data were expressed as the mean ± SD (*n* = 3). Compared with the control group, * *p* < 0.05 indicated a significant difference. Compared with the DON group, ## *p* < 0.01 indicated a very significant difference.

In order to detect the intervention effect of *Bacillus amyloliquefaciens* B10 on DON in promoting PCV2 infection, this study detected the main structural protein CAP of PCV2 according to the Western blot assay. After adding DON to PCV2-infected cells, the expression content of the CAP protein was extremely raised, and the expression level of the CAP protein in the *Bacillus amyloliquefaciens* B10 group was highly decreased. This result indicated that *Bacillus amyloliquefaciens* B10 itself could enhance the immune response. The expression of the CAP protein was also exceedingly diminished after pretreatment with *Bacillus amyloliquefaciens* B10, indicating that *Bacillus amyloliquefaciens* B10 alleviated PCV2 infection upregulated by DON (Figure 3).

### 2.4. Effect of DON on the LC3B Protein and Gene after Bacillus amyloliquefaciens B10 Pretreatment

**Figure 4:**
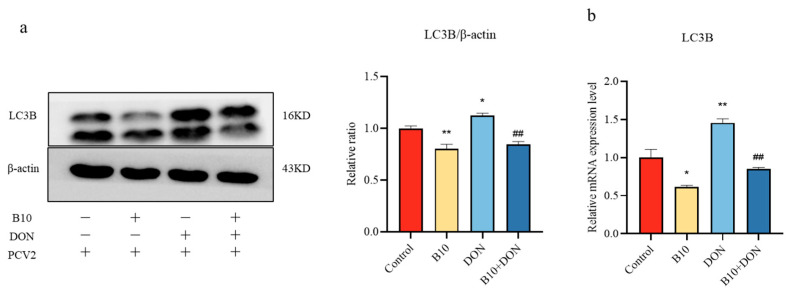
Effect of DON on the LC3B protein and gene expression after *Bacillus amyloliquefaciens* B10 pretreatment. (**a**) The expression of the LC3B protein was detected by the Western blot assay; (**b**) The expression of LC3B mRNA was detected by qPCR. The data for each assay were analyzed using a one-way analysis of variance (ANOVA). The data were expressed as the mean ± SD (*n* = 3). Compared with the control group, * *p* < 0.05 indicated a significant difference and ** *p* < 0.01 indicated a very significant difference. Compared with the DON group, ## *p* < 0.01 indicated a very significant difference.

In order to explore whether *Bacillus amyloliquefaciens* B10 alleviated DON-upregulated PCV2 infection through autophagy, the protein and gene expression of LC3B were determined by the Western blot assay and a quantitative PCR. The addition of DON significantly increased the expression level of LC3B, while the expression level of the LC3B protein in the *Bacillus amyloliquefaciens* B10 group and the *Bacillus amyloliquefaciens* B10 pretreatment group decreased significantly (Figure 4a,b). Thus, it was found that DON could promote the infection of PCV2 and reduce immune function through autophagy, while *Bacillus amyloliquefaciens* B10 could intervene in the role of DON by inhibiting autophagy.

### 2.5. Type I Interferons IFN-α, IFN-β mRNA Expression after Bacillus amyloliquefaciens B10 Pretreatment

**Figure 5:**
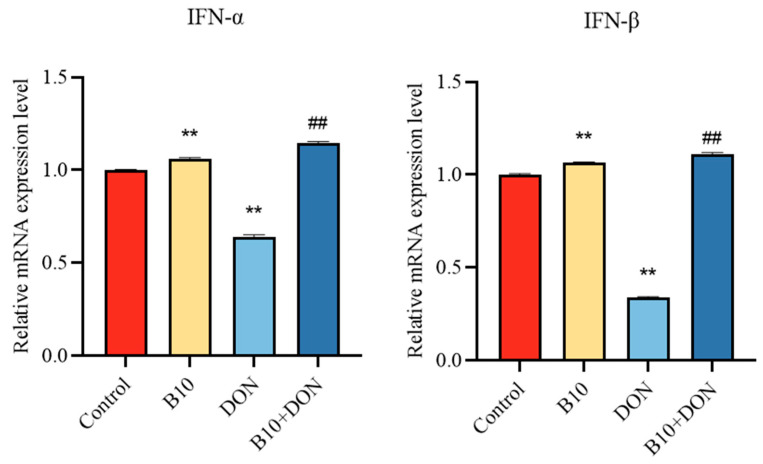
Effect of the DON on IFN-α and IFN-β mRNA expression levels after *Bacillus amyloliquefaciens* B10 pretreatment. The data for each assay were analyzed using a one-way analysis of variance (ANOVA). The data were expressed as the mean ± SD (*n* = 3). Compared with the control group, ** *p* < 0.01 indicated a very significant difference. Compared with the DON group, ## *p* < 0.01 indicated a very significant difference.

The above results showed that *Bacillus amyloliquefaciens* B10 could alleviate the infection of PCV2, which was upregulated by DON. The effect of *Bacillus amyloliquefaciens* B10 on the expression of type I interferon was investigated by detecting the mRNA expression levels of IFN-α and IFN-β. The study found that the IFN-α and IFN-β mRNA expression levels in the DON group decreased significantly, while the addition of *Bacillus amyloliquefaciens* B10 pretreatment significantly enhanced the IFN-α and IFN-β mRNA expression levels (Figure 5).

### 2.6. Expression of the Antiviral Proteins OAS and MX1 mRNA after Bacillus amyloliquefaciens B10 Pretreatment

**Figure 6:**
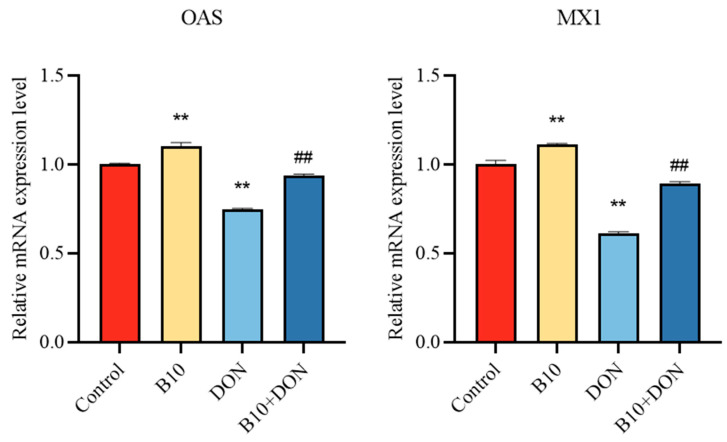
Effect of DON on the expression levels of the antiviral proteins OAS and MX1 mRNA after *Bacillus amyloliquefaciens* B10 pretreatment. The data for each assay were analyzed using a one-way analysis of variance (ANOVA). The data were expressed as the mean ± SD (*n* = 3). Compared with the control group, ** *p* < 0.01 indicated a very significant difference. Compared with the DON group, ## *p* < 0.01 indicated a very significant difference.

Type I interferons play a role by passing signaling factors to downstream antiviral proteins, which produce antiviral effects, thereby inhibiting viral infection. The above results showed that *Bacillus amyloliquefaciens* B10 could alleviate the inhibition of type I interferon induced by DON. The effect of *Bacillus amyloliquefaciens* B10 on antiviral proteins was further investigated, and the mRNA of the antiviral proteins OAS and MX1 was detected by qPCR. It was found that the antiviral proteins OAS and MX1 mRNA decreased significantly compared to the control group. However, for the DON group, the expression level of the *Bacillus amyloliquefaciens* B10 pretreatment group was significantly higher (Figure 6).

### 2.7. Expression of Immune Cytokine IL-10 mRNA after Bacillus amyloliquefaciens B10 Pretreatment

**Figure 7:**
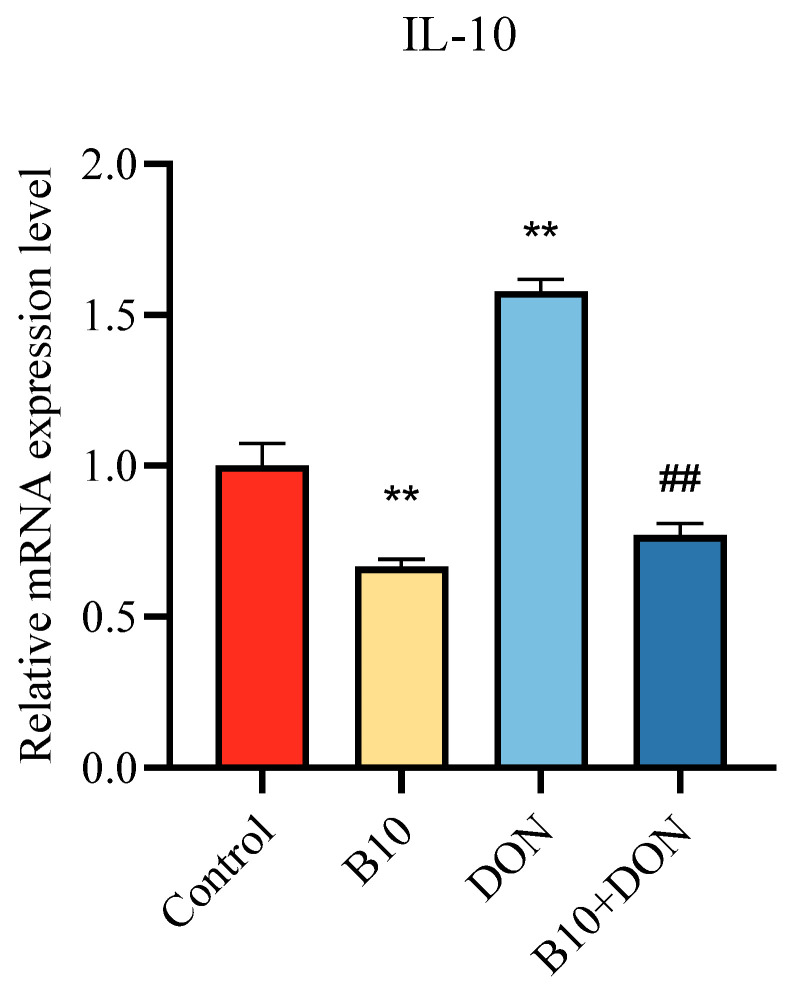
Effect of DON on the IL-10 mRNA expression level after *Bacillus amyloliquefaciens* B10 pretreatment. The data for each assay were analyzed using a one-way analysis of variance (ANOVA). The data were expressed as the mean ± SD (*n* = 3). Compared with the control group, ** *p* < 0.01 indicated a very significant difference. Compared with the DON group, ## *p* < 0.01 indicated a very significant difference.

Interleukin-10 (IL-10) is an immunosuppressive cytokine that plays a significant part in immunoreaction. In order to explore the effect of pretreatment with *Bacillus amyloliquefaciens* B10 on the immune factors, the expression level of IL-10 mRNA was detected by qPCR. Both the *Bacillus amyloliquefaciens* B10 and *Bacillus amyloliquefaciens* B10 pretreatment groups significantly reduced the expression of mRNA in IL-10, while the DON group significantly enhanced its expression (Figure 7).

### 2.8. Bacillus amyloliquefaciens B10 Pretreatment Relieves the Inhibitory Effect of DON on the cGAS–STING Signaling Pathway

**Figure 8:**
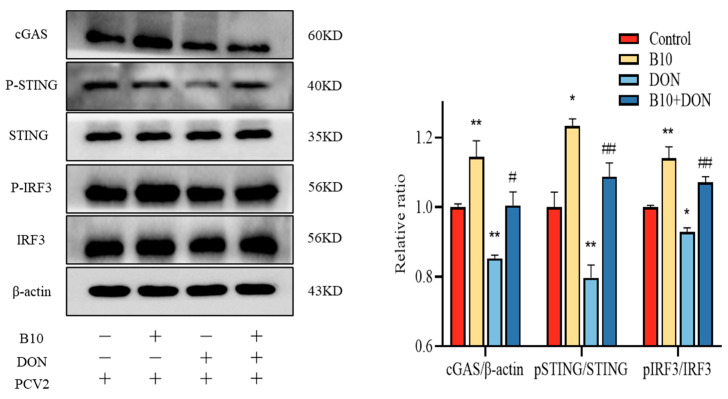
Effect of DON on the cGAS–STING signaling pathway after *Bacillus amyloliquefaciens* B10 pretreatment. The data for each assay were analyzed using a one-way analysis of variance (ANOVA). The data were expressed as the mean ± SD (*n* = 3). Compared with the control group, * *p* < 0.05 indicated a significant difference and ** *p* < 0.01 indicated a very significant difference. Compared with the DON group, # *p* < 0.05 indicated a significant difference and ## *p* < 0.01 indicated a very significant difference.

The CGAS–STING signaling pathway is one of the crucial pathways that controls the expression of antiviral proteins by regulating type I interferons, thereby exerting its role. In this assay, the expression of pathway proteins and downstream proteins was detected by the Western blot. The expression contents of cGAS, P-STING, and P-IRF3 were extremely raised in the *Bacillus amyloliquefaciens* B10 group, while the DON group was significantly reduced. Compared with the DON group, the expression content of cGAS, P-STING, and P-IRF3 in the *Bacillus amyloliquefaciens* B10 pretreatment group were extremely increased. Both the STING and IRF3 expressions were unchanged in the experiment (Figure 8).

## 3. Discussion

**Figure 9:**
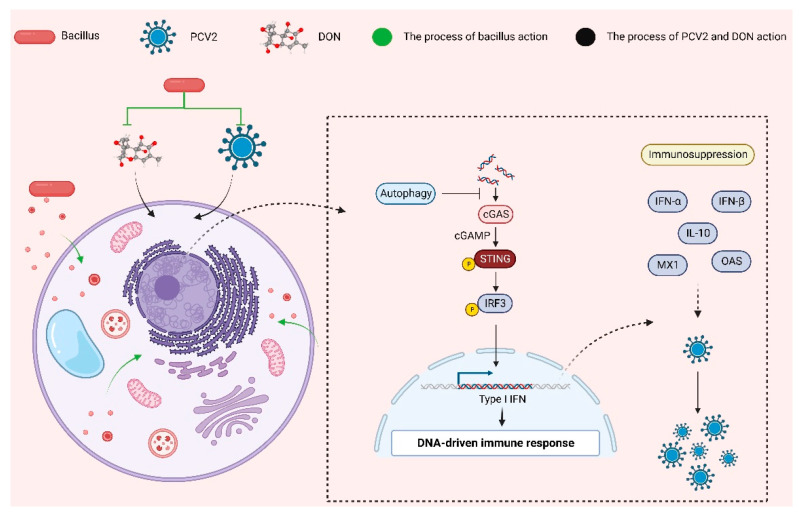
*Bacillus amyloliquefaciens* B10 alleviates the immunosuppressive effects of DON and PCV2 infection.

Feed and water sources consumed by animals are often contaminated with mycotoxins. The Food and Agriculture Organization of the United Nations (FAO) has found that up to 25% of products worldwide are polluted to some extent by mycotoxins [22]. However, among these mycotoxins, DON is the most common [23]. Pigs are sensitive to DON and are highly susceptible to DON contamination in pig feed. PCV2 is a globally transmitted virus that poses significant economic problems for the global pig industry [22]. PCV2 has been found to be replicated in lymphocytes, macrophages, and porcine tracheal epithelial cells, and DON has a regulatory effect on it [6,24]. However, DON can promote not only the replication of PCV2, but also the replication of other viruses. DON promotes the replication of porcine reproductive and respiratory syndrome virus (PRRSV) [25], increases the proliferation of viral hemorrhagic sepsis virus (VHSV) in rainbow trout gill epithelial cell lines [26], and upregulates the susceptibility of horses to equine herpesvirus type 1 (EHV1) [27]. In addition, DON can impair intestinal and respiratory resistance to reovirus (REO) infection in mice [28] and promote the proliferation of porcine epidemic diarrhea virus in intestinal cells [29]. It was found that the immunosuppressive effect of DON on the body can aggravate the infection of other pathogenic microorganisms. Therefore, it is of great significance to study the mechanism of immunosuppression induced by DON. This study was the first to link B10 to the immunosuppressive effects of infection with PCV2 and DON and found that B10 plays a significant part in detoxification and antiviral activity (Figure 9). The main new findings of this study were that (1) B10 could significantly interfere with PCV2 infection in vitro, which was related to the inhibition of DON on the cGAS–STING signaling pathway and type I interferon, and (2) that B10 could alleviate the immune-lowering effect of PCV2 and DON infection through autophagy.

Previously, antibiotics were commonly used to protect against certain bacterial and viral diseases. However, the persistent high incidence of antibiotic-resistant diseases has become a major global health threat [30]. In recent years, the regulatory role of microbial flora has been increasingly applied to actual production. The microbial flora can influence the physiological function of the organism through its colonization and metabolic activity [31]. Bacillus has been found to alleviate non-alcoholic fatty liver disease and inhibit undesirable obesity in mice [32]. *Lactobacillus plantarum* is used as a feed additive to protect intestinal function in mice and to prevent DNA breakage in the body [33]. *Bacillus amyloliquefaciens* B10 was able to improve the intestinal barrier, regulate the intestinal microbiota in mice, and relieve cecal inflammation [16]. As a result, this microorganism has been found to be an effective therapeutic tool for various diseases [34]. The main mechanisms by which probiotics help to defend the host from pathogenic infection are strengthening the epithelial barrier, enhancing adhesion to the intestinal mucosa, competitively inhibiting the adhesion of pathogens, synthesizing antimicrobial substances, producing secondary metabolites, modifying toxins and toxin receptors, and stimulating specific and non-specific immune responses to pathogens [35]. There are many studies on the detoxification of mycotoxins and the improvement of the body’s immune function by *Bacillus*. On the one hand, *Bacillus* strengthens the intestinal barrier and improves intestinal function by maintaining the balance of intestinal flora. On the other hand, chemicals secreted through their own transformation are at work. Bacillus is fed to piglets as a feed additive. It produces a large number of extracellular enzymes, which are not only closely correlated with digestion and absorption, but also promote feed intake, growth, and development in pigs [36]. The most direct method of pathogen inhibition by bacillus is the production of secondary metabolites. Metabolites such as antimicrobial proteins, lipopeptides, amino acids, nucleic acids, and polyketones all have biological functions. In particular, the antimicrobial effect of small molecular weight lipopeptides is highly significant [37]. This study found that DON can significantly increase the expression content of the CAP protein and promote in vitro replication of PCV2. However, *Bacillus amyloliquefaciens* B10 can effectively reduce the in vitro replication of PCV2, inhibit the promotion effect of DON on PCV2 infection, enhance the production of type I interferon (IFN-α, IFN-β) and antiviral proteins (OAS, MX1), relieve the immunosuppressive effect, and exert immune function.

In this study, the effect of DON on the production of the type I interferons IFN-α, IFN-β and antiviral proteins OAS, MX1, and related mechanisms after PCV2 infection was investigated. It was found that the exposure of DON significantly inhibited the expression of type I interferon and antiviral proteins, and the inhibitory effect of DON was related to the inhibition of the cGAS–STING signaling pathway. DON inhibited the transcriptional activation of type I interferon by blocking the nucleation of this pathway and its downstream interferon regulator IRF3. Since its discovery, the cGAS–STING pathway has been a research hotspot and has attracted the attention of many researchers. More and more studies have found that the cGAS–STING pathway plays an important role in anti-infection, antitumor, and autoimmune diseases. cGAS acts as a DNA recognition receptor and DNA receptor that recognizes self and non-self DNA, inducing innate immunity. At present, there have been many reports that the cGAS–STING pathway is closely related to DNA virus infection. The cGAS–STING pathway can recognize a variety of viruses, such as poxvirus (VACV), human papillomavirus (HPV), human cytomegalovirus (HCMV), adenovirus (Ad), herpes simplex virus (HSV-1), etc., and is activated early in infection to induce innate immune-initiated type I interferon activation, which can be eliminated in time to protect the body [38,39,40].

In addition, the role of autophagy in DON causing co-infection was explored. LC3 is a marker protein of autophagosome, and the LC3-II expression represents the formation of autophagosome. In this study, DON exposure significantly increased the LC3II expression and induced autophagy. However, *Bacillus amyloliquefaciens* B10 can inhibit the adverse autophagy reaction caused by DON, relieve the inhibitory effect of DON on the cGAS–STING signaling pathway, restore the production of type I interferon and other immune factors, and enhance the body’s natural immune function, thereby inhibiting the toxic effect of DON and hindering PCV2 infection. There are many reports about the involvement of autophagy in virus replication, including the activation of the P38/MAPK and JAK2/STAT3 signaling pathways and the inhibition of the PI3K/Akt signaling pathway to induce autophagy [41,42,43]. In addition to autophagy, many mechanisms by which probiotics regulate immunity have been reported. *Bacillus amyloliquefaciens* can alleviate the liver damage caused by aflatoxin to the mouse liver by improving oxidative stress and apoptosis [15]. In addition, *Bacillus amyloliquefaciens* can also activate the TLR4/MyD88 signaling pathway as well as the NF-κB pathway, release immune cytokines, and increase the expression of co-adhesion molecules. Eventually, it has the ability to activate immune responses to pathogen infection [44].

Further studies should be carried out in animals to investigate whether *Bacillus amyloliquefaciens* B10 can alleviate the toxicity of PCV2 and DON infection in vivo. It was concluded that *Bacillus amyloliquefaciens* B10 could not only alleviate the immunosuppression induced by PCV2 and DON but also prevent and treat the host damage caused by the combined infection of both. This provides a new target and direction for the prevention of diseases caused by DON and PCV2 co-infection and its mechanism of action. It lays a research foundation for the application of *Bacillus amyloliquefaciens* B10 probiotics in feed addition and food health care.

## 4. Conclusions

*Bacillus amyloliquefaciens* B10 not only enhances the host immune response but also interferes with the immunosuppression induced by DON and PCV2 to restore host resistance. In this study, it was confirmed that the antiviral effect of *Bacillus amyloliquefaciens* B10 restored the expression of type I interferon and antiviral proteins by relieving the inhibition of DON on the cGAS–STING signaling pathway, blocking the promotion of DON on PCV2 infection and the toxicity of both in the host. At the same time, this study found that the intervention of *Bacillus amyloliquefaciens* B10 was produced by inhibiting autophagy.

## 5. Materials and Methods

### 5.1. Chemicals and Virus

DON was bought from Sigma-Aldrich (St Louis, MO, USA) and diluted using a sterile PBS. DON at a concentration of 1 μg/mL measured by the MTT assay was used for subsequent testing. PCV2 and the PK-15 cells were derived from the Department of Veterinary Infectious Diseases and Veterinary Internal Medicine, Nanjing Agricultural University, respectively. PCV2 was amplified by the PK-15 cells, and the viral titer was 10^6^ TCID50/_0.1_ mL measured by the indirect immunofluorescence assay (IFA). The PK-15 cells were cultured in Dulbecco’s Modified Eagle Medium (DMEM) (Gibco, Shanghai, China) containing 10% fetal bovine serum and a 1% penicillin-streptomycin solution at 37 °C, 5% CO_2_.

### 5.2. Design of Experiments

First, the optimal effective concentration and treatment time of *Bacillus amyloliquefaciens* B10 were first explored. Second, the effect of *Bacillus amyloliquefaciens* B10 on the combined toxicity of PCV2 and DON was investigated. The PK-15 cells infected with PCV2(100 MOI) were used as the experimental model, and the experiments were divided into four groups (the control, *Bacillus amyloliquefaciens* B10 (2.1 × 10^6^ cfu/mL), DON (1 μg/mL), *Bacillus amyloliquefaciens* B10 + DON) and treated with *Bacillus amyloliquefaciens* B10 for 12 h and DON for 24 h.

### 5.3. Cell Viability Assay

A density of 3 × 10^3^ cells/well were evenly packed into 96-well plates. The cells were treated according to the experimental design and the cell viability was determined using the MTT test method. After the treatment was completed, 20 μL of the MTT solution at a concentration of 5 mg/mL was added to each well protected from light and incubated in an incubator for 4 h followed by 150 μL of DMSO per well. The violet crystals were dissolved using a shaker, and finally the absorbance value was determined at 490 nm using an enzyme-labeled instrument.

### 5.4. qPCR Analysis

The processed sample was collected using the RNA isolater Total RNA Extraction Reagent to extract the total RNA. cDNA was then obtained using a reverse transcription kit and stored at −20 °C. A qPCR was performed using the ChamQ Universal SYBR qPCR Master Mix, and all the reagents were purchased from Vazyme Corporation (Nanjing, China). The primers were designed by Sangon Biotech (Shanghai, China), and the specific primers are shown in Table 1. The ΔCycle threshold (ΔCt) method was used to determine the relative expression.

### 5.5. Western Blot

First, the PK-15 cells were lysed with RIPA buffer, and after the cells were disrupted, the total protein of the cells was collected. The concentration of the total protein in the sample was determined using the BCA Protein Quantification Kit (Vazyme, Nanjing, China), and the total proteins were prepared using the SDS-PAGE Protein Loading Buffer (5×) (Biosharp, Nanjing, China). Second, gels were made using the 12% One-Step PAGE Gel Fast Preparation Kit (Vazyme, Nanjing, China) to separate the total proteins, and the proteins of different sizes were transferred to the PVDF membrane using Bio-Rad Trans-Blot^®^ Turbo™ (Hercules, CA, USA). Third, the PVDF membranes were blocked in 5% skim milk for 1 h at ambient temperature and washed using a TBS solution containing 0.05% Tween-20 for 10 min. The primary antibody was diluted using a primary antibody dilution, and the PVDF membranes were incubated with the primary antibody at 4 °C for 16–18 h. Subsequently, it was incubated with an HRP-conjugated secondary antibody for 1 h at ambient temperature. Finally, LC3II, CAP, cGAS, STING, IRF3, and other proteins were determined according to the Western blot assays. Information on the use of these antibodies is shown in Table 2 (cGAS, STING, P-STING, IRF3, P-IRF3, CAP, LC3A/B, 1:1000; β-Actin, 1:10,000).

### 5.6. Statistics

All the statistical analyses were analyzed using IBM SPSS Statistics 26.0, either using a one-way ANOVA to detect significant differences between the treatment groups, grayscale analysis of the protein bands using ImageJ-win64 (National Institutes of Health, Bethesda, MD, USA), mRNA CT analysis using QuantStudio™ Real-Time PCR Software (version 6) (Applied Biosystems, New York, NY, USA), or plotting using GraphPad Prism 8.1 (San Diego, CA, USA). The data were expressed as the mean ± SD (*n* = 3). Compared with the control group, * *p* < 0.05 indicated a significant difference and ** *p* < 0.01 indicated a very significant difference. Compared with the DON group, # *p* < 0.05 indicated a significant difference and ## *p* < 0.01 indicated a very significant difference.

## Figures and Tables

**Table 1:** Primer sequences for the qPCR.

Primer	Primer Sequences	Tm
GAPDH	F: CGTCAAGCTCATTTCCTGGT R: TGGGATGGAAACTGGAAGTC	55.2 54.5
LC3	F: CCGAACCTTCGAACAGAGAG R: AGGCTTGGTTAGCATTGAGC	55.5 55.6
IFN-α	F: CAACCAGGTCCAGAAGGCTCAAG R: GCTGATCCAGTCCAGTGCAGAAC	56.5 56.5
IFN-β	F: CAGTATTGATTATCCACGAGA R: TCTGCCCATCAAGTTCCAC	48.3 55.1
OAS	F: CCTTGCTGCTATTCCGTGCTCTG R: TCCTGTTGTGGCTTAGAGACCTCTC	56.5 52.0
MX1	F: CGGCTGTTTACCAAGATGCGAAATG R: TTCACAAACCCTGGCAACTCTCTC	48.0 50.0
IL-10	F: AAGCCTTGTCAGAGATGATCCAGT R: CTCCTTGATATCCTCCCCATCACTC	45.8 52.0

Note: GAPDH: glyceraldehyde-3-phosphate dehydrogenase (NM_001206359.1); LC3: glyceraldehyde-3-phosphate dehydrogenase (NM_001190290.1); IFN-α: interferon α(NM_001166318.1); IFN-β: interferon β (NM_001003923.1); OAS: oligoadenylate synthetase 2′-5′ (NM_214303.2); MX1: myxovirus resistance 1 (NM_214061.2); IL-10: interleukin-10 (NM_214041.1).

**Table 2:** Antibody information.

Antibody	Number
cGAS Rabbit pAb	A8335
STING Rabbit mAb	A3262
Phospho-STING Rabbit mAb	AP1223
IRF3 Rabbit pAb	A11118
Phospho-IRF3-S386 Rabbit pAb	Ap0857
β-Actin Rabbit pAb	Ac006
Rabbit anti-PCV2 Cap protein pAb	Bs20021R
LC3A/B (D3U4C) Rabbit mAb	#12741

## Data Availability

Data are contained within the article.
